# Supplementary material for: Regulatory Control of Rishirilide(s) Biosynthesis in Streptomyces bottropensis
Source: Microorganisms. 2021 Feb 12;9(2):374. doi: 10.3390/microorganisms9020374 (PMC7917814; doi:10.3390/microorganisms9020374)
Supplement: Supplementary file 1 [file microorganisms-09-00374-s001.pdf]

Supplementary Materials

Regulatory control of rishirilide(s) biosynthesis in *Streptomyces bottropensis*

Olga Tsypik [1], Roman Makitrynskyy [1], Xiaohui Yan [1], Hans-Georg Koch [2], Thomas Paululat [3] and Andreas Bechthold [1,*]

[1] Institute for Pharmaceutical Biology and Biotechnology, Albert-Ludwigs-Universität, Stefan-Meier-Straße 19, 79104 Freiburg, Germany.
[2] Institute for Biochemistry and Molecular Biology, ZBMZ, Faculty of Medicine, Albert-Ludwigs-Universität, Stefan-Meier-Straße 17, 79104 Freiburg, Germany
[3] Organic Chemsitry II, Universität Siegen, Adolf-Reichwein-Strasse 2, 57068 Siegen, Germany
* Correspondence: andreas.bechthold@pharmazie.uni-freiburg.de

Table of Contents

Table S1. Strains and plasmids used in this work ... 3

Table S2. Primers List ... 5

Figure S1. Transcriptional profile of regulatory genes in the wild type strain and in different mutants. ... 8

Figure S2. Domain organization and sequence of RslR3 ... 9

Figure S3. Sequences alignment of RslR4 versions ... 10

Generation of *S. bottropensis* Δ*rslO7* ... 11

Figure S4. LC-MS analysis of metabolic compounds produced by *S. bottropensis* (A) and *S. bottropensis* Δ*rslO7* (B) ... 11

Isolation and purification of compounds RSHO7a, RSHO7b and RSHO7d from *S. bottropensis* Δ*rslO7* ... 12

NMR data for compounds ... 12

NMR data of compound RSH-O7a ... 13

NMR data of compound RSH-O7b ... 14

NMR data of compound RSH-O7d ... 15

^{1}H NMR of RSH-O7a ... 16

^{13}C NMR of RSH-O7a ... 16

COSY of RSH-O7a ... 17

HSQC of RSH-O7a ... 17

HMBC of RSH-O7a ... 18

^{1}H NMR of compound RSH-O7b 19
^{13}C NMR of compound RSH-O7b 19
COSY NMR of compound RSH-O7b 20
HSQC NMR of compound RSH-O7b 20
HMBC NMR of compound RSH-O7b 21
^{1}H NMR of compound RSH-O7d 22
^{13}C NMR of compound RSH-O7d 22
COSY NMR of compound RSH-O7d 23
HSQC NMR of compound RSH-O7d 23
HMBC NMR of compound RSH-O7d 24
References 25

Table S1. Strains and plasmids used in this work**.**

Strain / Plasmid	Description	Reference
Streptomyces		
S. bottropensis	Wild type, rishirilides producer	[1]
S. bottropensis ΔR1	mutant with in frame replacement of *rslR1* with spectinomycin resistance cassette	This work
S. bottropensis ΔR2	mutant with in frame replacement of *rslR2* with spectinomycin resistance cassette	This work
S. bottropensis ΔR3	mutant with in frame replacement of *rslR3* with spectinomycin resistance cassette	This work
S. bottropensis ΔR4	mutant with in frame replacement of *rslR4* with spectinomycin resistance cassette	This work
S. bottropensis ΔR1 pSET-rslR1	*S. bottropensis* ΔR1 carrying pSET-rslR1	This work
S. bottropensis ΔR2 pSET-rslR2	*S. bottropensis* ΔR2 carrying pSET-rslR2	This work
S. bottropensis ΔR3 pSET-rslR3	*S. bottropensis* ΔR3 carrying pSET-rslR3	This work
S. bottropensis ΔR3 pTES-rslR3	*S. bottropensis* ΔR3 carrying pTES-rslR3	This work
S. bottropensis pUWLH-rslR1	*S. bottropensis* carrying pUWLH-rslR1	This work
S. bottropensis pUWLH-rslR2	*S. bottropensis* carrying pUWLH-rslR2	This work
S. bottropensis pUWLH-rslR3	*S. bottropensis* carrying pUWLH-rslR3	This work
S. bottropensis pUWLH	*S. bottropensis* carrying pUWLH	This work
S. bottropensis pGUS-R4p	*S. bottropensis* carrying pGUS-R4p	This work
S. bottropensis ΔR4 pGUS-R4p	*S. bottropensis* ΔR4 carrying pGUS-R4p	This work
S. bottropensis pGUS-T4p	*S. bottropensis* carrying pGUS-T4p	This work
S. bottropensis ΔR4 pGUS-T4p	*S. bottropensis* ΔR4 carrying pGUS-R4p	This work
S. bottropensis pGUS	*S. bottropensis* carrying pGUS	This work
S. bottropensis pGUS-R1p	Wild type carrying pGUS-R1p	This work
S. bottropensis pGUS-R2p	Wild type carrying pGUS-R2p	This work
S. bottropensis ΔR1 pGUS-R1p	ΔR1 carrying pGUS-R1p	This work
S. bottropensis ΔR2 pGUS-R1p	ΔR2 carrying pGUS-R1p	This work
S. bottropensis ΔR3 pGUS-R1p	ΔR3 carrying pGUS-R1p	This work
S. bottropensis ΔR1 pGUS-R2p	ΔR1 carrying pGUS-R2p	This work
S. bottropensis ΔR2 pGUS-R2p	ΔR2 carrying pGUS-R2p	This work
S. bottropensis ΔR3 pGUS-R2p	ΔR3 carrying pGUS-R2p	This work
Escherechia coli		
XL1Blue	general cloning host	Agilent
ET12567 (pUZ8002)	host used for *E.coli-Streptomyces* intergeneric conjugation, methylation deficient	[2]
BW25113 (pIJ790)	host for recombineering experiments	[2]

BL21 (DE3) StarTM	host for protein production	Thermo Fisher Scientific
**Plasmids**		
pET28a(+)	cloning vector for His-tagged protein production in *E. coli*, kanamycin resistance	Novagen
pET28a-rslR3	pET28a derived plasmid for production of His-tagged RslR3	This work
pET28a-rslR3DBD	pET28a derived plasmid for production of His-tagged RslR3DBD	This work
pET28a-rslR3DBDP	pET28a derived plasmid for production of His-tagged RslR3DBDP	This work
pET28a-rslR4	pET28a derived plasmid for production of His-tagged RslR4	This work
pSET152	φC31-based integrative vector	[3]
pSET-rslR1	pSET152 carrying gene *rslR1* with its promoter region	This work
pSET-rslR2	pSET152 carrying gene *rslR2* with its promoter region	This work
pSET-rslR3	pSET152 carrying gene *rslR3* with its promoter region	This work
pTES	pSET152 carrying *ermE*p	[4]
pTES-rslR3	pTES carrying *rslR3*	This work
pUWLH	Replicative vector for gene overexpression, carrying ermEp	[5]
pUWLH-rslR1	pUWLH carrying gene *rslR1*	This work
pUWLH-rslR2	pUWLH carrying gene *rslR2*	This work
UWLH-rslR3	pUWLH carrying gene *rslR3*	This work
pGUS	Promoter probe vector; pSET152 carrying reporter gene *gusA*	Myronovskyy
pGUS-R1p	pGUS carrying fused *gusA* with *rslR1* promoter region	This work
pGUS-R2p	pGUS carrying fused *gusA* with *rslR2* promoter region	This work
pGUS-R3p	pGUS carrying fused *gusA* with *rslR3* promoter region	This work
pGUS-R4p	pGUS carrying fused *gusA* with *rslR4* promoter region	This work
pGUS-T4p	pGUS carrying fused *gusA* with *rslT4* promoter region	This work
pBluescriptIIKS$^+$	General purpose cloning vector. In this work used as a template for amplification of ampicillin resistance gene.	MBI Fermentas
pCDFDuet	cloning vector for His-tagged protein production in *E. coli*. In this work used as a template for amplification of spectinomycin resistance gene.	Merk
cos4-int::bla	cos4 carrying integrase gene (*int*) replaced by ampicillin resistance gene	This work

cos4-int::bla-rslR1::Sp	cos4-int::bla carrying *rslR1* replaced by spectinomycin resistance gene	This work
cos4-int::bla-rslR2::Sp	cos4-int::bla carrying *rslR2* replaced by spectinomycin resistance gene	This work
cos4-int::bla-rslR3::Sp	cos4-int::bla carrying *rslR3* replaced by spectinomycin resistance gene	This work
cos4-int::bla-rslR4::Sp	cos4-int::bla carrying *rslR4* replaced by spectinomycin resistance gene	This work

Table S2. Primers List

Primer Name	Sequence	Purpose
Int-aatP::amp-f	GGGGCTTCACGTTTTCCCAGGTCAGAAGCGG<br>TTTTCGGGA TTACAATTTAGGTGGCACTT	deletion of integrase gene
Int-aatP::amp-r	GCTGTGCGCCCGTCTCAGCGCCTAACAGGC<br>TTCCCGGGTG AGGATCTTCACCTAGATCCT	
rslR1-RedET-Fw	CCTGAACCACCGCCCCAGGCGAAGAGAGGAT<br>CCACCATGGCTAGCGGAGCGTAGCGACCGAGTG	spectinomycin cassette for *rslR1* gene deletion
rslR1-RedET-Rv	TCGCGTTGCAGAGGTACGGGCACGGGAGGGG<br>TCTCCTTA**GCTAGC**GGCTATTTAACGACCCTGC	
rslR2-RedET-Fw	GTGCCCGTAGCCCATCAAGCCAGCCCTGGAG<br>GCGAGATGGCTAGCGGAGCGTAGCGACCGAGTG	spectinomycin cassette for *rslR2* gene deletion
rslR2-RedET-Rv	GAGTCCGGCTCCACGCGTACGGGCCGGGCGC<br>GGGGGTCAGCTAGCGGCTATTTAACGACCCTGC	
rslR3-RedET-Fw	CGGCTGACCGTGCGCAGGGGGACCGACGGGG<br>CAGGTGTGGCTAGCGGAGCGTAGCGACCGAGTG	spectinomycin cassette for *rslR3* gene deletion
rslR3-RedET-Rv	TGCCCGAAGGCGCCCCTGTCACTGCTCCCGC<br>CACCGTCAGCTAGCGGCTATTTAACGACCCTGC	
rslR4-RedET-Fw	TATAGTGGAGGGGGAGTGAGCGAAGGGGAGG<br>CGGACATGGCTAGCGGAGCGTAGCGACCGAGTG	spectinomycin cassette for *rslR4* gene deletion
rslR4-RedET-Rv	CTTCACGGACAGGGCCGAAAGGAGGGGGTGA<br>CGCGCTTAGCTAGCGGCTATTTAACGACCCTGC	
rslR1-gus-f	GCTCTAGAGATGGATCAGCTGCGCGGT	construction of pSET-rslR1
rslR1MuDet-Fw	TGTCGCCGAGCAGCTTCAG	
rslR2-gus-f	CCTCTAGACACCCGCACTCTGGCCAT	construction of pSET-rslR2
rslR2MuDet-Fw	GCATGCCGGCGGCGATGTC	
rslR3-gus-f	GGTCTAGAGGAGGCCCGGCGGGTC	construction of pSET-rslR3
rslR3MuDet-Fw	AGGATTCGTGCGTACATC	
R3-XbaI-pTESa	AATCTAGACTGCCGAAGAATGTCCGGAA	construction of pTes-rslR3
rslR3MuDet-Fw	AGGATTCGTGCGTACATC	
msc24overExfw	CCCATCGATTCTCTTAAGGACCACGGAAGCCGCACC	construction of pUWLH-rslR1
msc24overExrv	GAACAGAAGCTTACGGCCGGCGCCGG	
msc30overExfw	ATTCCGAAGCTTCAAGCCAGCCCTGGAGG	construction of pUWLH-rslR2
msc30overExrv	AACACTGCAGAGCTAGCGGGGGTCAGCCGGCC	
msc31overExfw	TACGAATTCTCGCTAGCGGAGCGGACGGCCTG	construction of pUWLH-rslR3
msc31overExrv	GGACTAGTCACTGCTCCCGCCACCGT	
rslR1-gus-f	GCTCTAGAGATGGATCAGCTGCGCGGT	construction of pGUS-rslR1p
rslR1-gus-r	AAAGGTACCTGGGGCGGTGGTTCAGGT	
rslR2-gus-f	CCTCTAGACACCCGCACTCTGGCCAT	construction of pGUS-rslR2p
rslR2-gus-r	AAAGGTACCGCTGGCTTGATGGGCTACG	
rslR4-gus-f	CGTCTAGACGGCGAGCAGATAGCCG	construction of pGUS-rslR4p
rslR4-gus-r	GGGGTACCGCTCACTCCCCCTCCACTAT	
rslT4-gus-f	GCTATCTAGACGGTGGACAGCTCCTGCAC	construction of pGUS-rslT4p / EMSA
rslT4-gus-r	GCATGGTACCTGGAGCGGTCTGCGAGG	

RT-R1-f	AGATCTGGGGCGAGCATCC	RT-PCR
RT-R1-r	GTGCAGGGCTTCGTGGGT	
RT-R2-f	AATGCGTGGACGAACTGTGG	
RT-R2-r	GGAGAGGGCGATCATCAGCT	
RT-R3-3-f	GAGGTTGGACTGCTCGACGC	
RT-R3-3-r	TCGGCGACATGGCCTTCA	
RT-R4-f	GCTGATGGTCGGTCAGCTCA	
RT-R4-r	ACGAGGCGACATCGAGCAGT	
RT-hrdB-f	CGACTACACCAAGGGCTACAA	
RT-hrdB-r	TCGTCTTGGACTCGATCTGA	
RT-T1-f	GTCGTCATCAGGACGTCCAC	RT-PCR and EMSA
RT-T1-R	ACGAGGAATCCGCCATCTC	
RT-C1-f	AGCCACGCACGCAACACCG	
RT-C1-r	GACGCCTGAGTCCGGCATG	
RT-K12-f	AAGTCGTCGATGCTCAGTGC	
RT-K12-r	GCTGCTGGTCCTGCGTGAC	
RT-AK4-f	CCATACCGGGTTGTTGCG	
RT-AK4-r	GGCGATGTACAGGTCCTCGA	
RT-O12-f	CAGCCGAAGCGACTCCTCGA	
RT-O12-r	CACCACCTCAGCGCCGTC	
RT-O2P-f	TCCATCGACACGGAGGTGA	
RT-O2P-r	CGGCTGCTCGACGAGAGC	
RT-R1C2-f	GTGGAGCAGCAACAGTCCGA	
RT-R1C2-r	CCGTGTCCACGACGAGGA	
RT-C2O3-f	GACTGGTCGGCAACCTCCA	
RT-C2O3-r	CCACCACGACGGTGGCTC	
RT-C3R2-f	TCGGACACGGTGTGGGTGT	
RT-C3R2-r	CCGACGCCCTTGAGGTGTT	
RT-R23-f	TCGGAGAGGGTGAGCGTG	
RT-R23-r	CGGACGACAGCGGCAC	
RT-R3O6-f	GCTGCTTGGGGGACGAGAC	
RT-R3O6-r	TGGTGCTCTCCACGATCCAC	
RT-T4O7-f	TCAGTCTGATCGCTGCCCTC	
RT-RT4O7-r	ACCGCAGCTCGACGGTGA	
RT-O78-f	GGACCTGTCTCTCCTGCTCG	
RT-O78-r	TCTCGATGAAATTGACACCGA	
RT-O89-f	ACGGCGAGGTCGCGAAAGT	
RT-O89-r	GAGGAAGACGGCCGTGCTGA	
RT-O910-f	CGAACTGCTCGATGCGGAA	
RT-O910-r	CCGATGCCGCTCGTGCT	
RT-K3A-f	GGAGGCATCGAAGAGGGAGA	
RT-K3A-r	GGTCGCGAGAGGAGGCTC	
RT-T12-f	AGGAGCAGGGTGAACAGGGT	
RT-T12-r	TCGTCGTCACGCACGAGAT	
RT-T3O1-f	CCATCGCCAACAGGACCA	
RT-T3O1-r	CACCATCAGCCTCCAGGTCA	

RT-PR1-f	GAACGGGTGCCGAACCAGT	
RT-PR1-r	CGCGCTCGATGTCGCGT	
RT-O10H-f	ACGAAGTTCCAGGCGAAGAT	
RT-O10H-r	GACCGAGATCCACATGCTCA	
RT-O6R4-f	ACCTCGGCGACCTTCAGC	
RT-O6R4-r	GCTGATGGTCGGTCAGCTCA	
RT-O4O5-f	GACCGTACGAGGGAGTAACG	
RT-O4O5-r	CCCGCGGTTCTTCTACGA	
R4-NcoI-17aa	AAACCATGGCTCATGGAGTTGGTTATAG	construction of pET28a-rslR4 for his-tagged RslR4 production
R4-XhoI	AAACTCGAGAGGGGTGGCGGCTCCGGC	
R3s-NdeI-f	AATTAACATATGAATGGGGAACACGCTTC	construction of pET28a-rslR3 for his-tagged RslR3 production
R3-HindIII-r	ATAAAGCTTGCCACCGTCATGCACTGC	
R3s-NdeI-f	AATTAACATATGAATGGGGAACACGCTTC	construction of pET28a-rslR3DBD for his-tagged RslR3DBD production
RslR3-DBD-R	TATAAGCTTTCAGGTGGGCTGCACCC	
R3s-NdeI-f	AATTAACATATGAATGGGGAACACGCTTC	construction of pET28a-rslR3DBDP for his-tagged RslR3DBDP production
RslR3-DBDP-R	TATAAGCTTTCAGAGCAATCGGAAGAACCGTC	

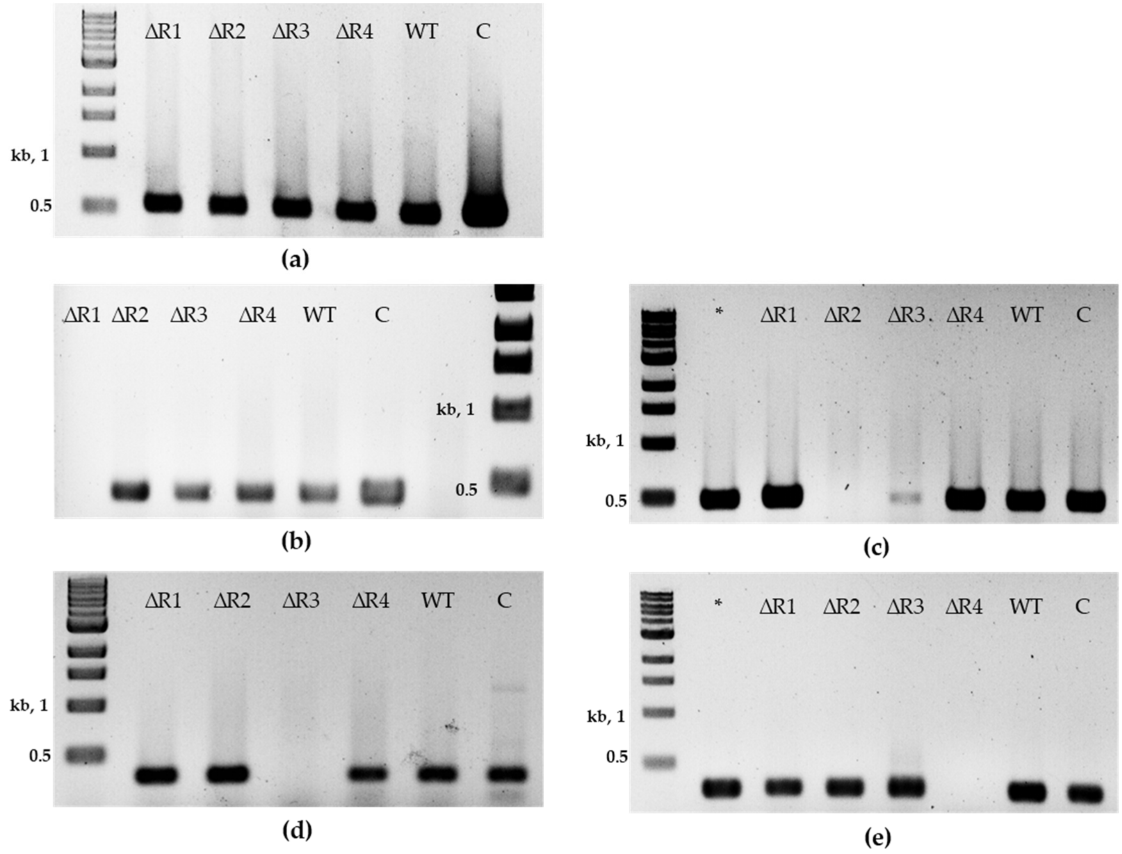

Figure S1. Transcriptional profile of regulatory genes in the wild type strain and in different mutants. Unprocessed agarose gels of sqRT-PCR analysis of (**a**) *hrdB*, (**b**) *rslR1*, (**c**) *rslR2*, (**d**) *rslR3* and (**e**) *rslR4* transcripts in *S. bottropensis* (WT), *S. bottropensis* ΔR1 (ΔR1), *S. bottropensis* ΔR2 (ΔR2), *S. bottropensis* ΔR3 (ΔR3), *S. bottropensis* ΔR4 (ΔR4). PCR was performed using cDNA and chromosomal DNA (C) as a template. * - sample that is not related to this work. Molecular marker is 1 kb Ladder (NEB.)

(a)

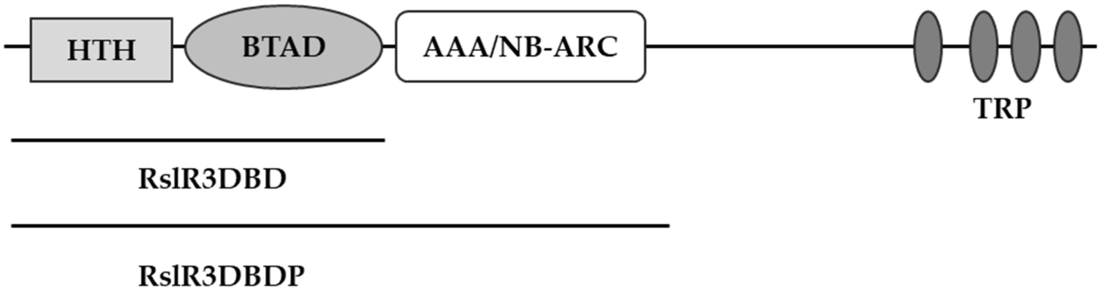

(b)

MWPGSHGGGPLRKHVLLWRWARLTVRRGTDGAGVHVNGEHASIYDFRILGPLAVLTGGRELTVSSPKQRVLLASL
LLRADRVVTSDELIRRLWDDQPPRDTRAALHVHTTRLRALLRESLDVGDPPVIETHPGGYRLAMDSAALDLTRYRK
AADEARLARGRGDTARELAALERALAEWRGDPFETVPSASLQTEAVPQLAEERLTLVERRFALLLDLGGSADIIPQL
RALAAEHPVRERLREYLMRALHRTGQVAAALEEYRDYYRMLDRELGLPPGQELQELHRQLLAETGELHTPQAGPS
GRRAYAVREVHRTAGVQPTNPSAPWVVQRQLPPEATHFVGREELLEEVVAALTPRQRAAVPLVSLVGPPGVGKTA
LALRAAHRLSPAYPDGQWYIRLHDACGGARPVHDIIAELLHASGADVSVLPTERHQLTGVLRSRLADRRVLIVIDD
VNNSEQVPDLLPGSPSCAVLALHREYRPDLVAVYGARSFTLDLLSQKEGEQVLTAVLGRAPETLCATAVTELVELC
GRLPLALRIAAGHLAGRPWRSVESFAEALREGDPLELLALGDSPSTAVRAAFGVAYESLPALSRRFFRLLGVVGDMA
FTALTAAQLADCEPQVADQLLDKLAAAQLIEVSSQDTYRFHSLIALYAAERSIEEDGPADRRQALNRLCRWYLRRT
DEAVRSCYPGFLRVFRPDPHGPDIEVEPRAAQEWLRVEQSNLVALVVRAADEHLDEVCVRLVDMLRGYFTLGRLQ
TDWLTIAQAGLRAARSLGDQRATAVMRLSVGLALQGLNRLPDAARELRLAHREFIRLGVRDFEAVTVNAIAMNQ
LQRPTKHIDSAVTLLERGLEISRHLELRHVEARGLMYLGMARHSQGRLHVAEAHFSRAAAILDEDGVQRSRPEVLA
RLGAVHADLREWDAAMANLSLALSLSKEFNAPHSTVLASYGLAQVYACNSYVDLAYQHAESAVVIARDHGYVAL
EANARNTLGGLHLIRGRSADAREEFTRTLAIAERIGHPQSEAHALIGLGRLELAGGRPAEAYQLGARAVTVADGSG
LLLLRAQAEELCRRTGNTPGREHPESAGRRADDSGTVLPEASGSLHALFPECSA

Figure S2. Domain organization and sequence of RslR3. (**a**) RslR3 consist of helix-turn-helix DNA-binding domain (HTH), Bacterial Transcriptional Activation (BTAD), ATPase (AAA/NB-ARC) and tetratricopeptide repeat (TPR) domains. RslR3DBD and RslR3DBDP are truncated versions of RslR3, that include HTH, BTAD, and additionally ATPase domains, respectively. (**b**) Amino acid sequence of RslR3. Highlighted is sequence of RslR3DBD used in this work.

```
RslR4-17aa      MHGVGYSGGGVSEGEADMAADDRGGRPGEFDLWRRMTLMVGQLNQSLEKTLASEHQISLP 60
RslR4-7aa       ----------VSEGEADMAADDRGGRPGEFDLWRRMTLMVGQLNQSLEKTLASEHQISLP 50
RslR4           -----------------MAADDRGGRPGEFDLWRRMTLMVGQLNQSLEKTLASEHQISLP 43
                                 *******************************************

RslR4-17aa      ELMVLIELRHGHERGTRVQELSTAVGLDQSSMSRLVTRLENKGLTTRVSCEHDRRGVYCM 120
RslR4-7aa       ELMVLIELRHGHERGTRVQELSTAVGLDQSSMSRLVTRLENKGLTTRVSCEHDRRGVYCM 110
RslR4           ELMVLIELRHGHERGTRVQELSTAVGLDQSSMSRLVTRLENKGLTTRVSCEHDRRGVYCM 103
                ************************************************************

RslR4-17aa      LTPAGAERGERAEARCREELTGLLDVASFDDRWASLVSRFRHSAAGAATP  170
RslR4-7aa       LTPAGAERGERAEARCREELTGLLDVASFDDRWASLVSRFRHSAAGAATP  160
RslR4           LTPAGAERGERAEARCREELTGLLDVASFDDRWASLVSRFRHSAAGAATP  153
                **************************************************
```

Figure S3. Sequences alignment of RslR4 versions.

Analysis of the DNA region encoding RslR4 identified another two putative translational start point resulting in proteins with 7 and 17 extra amino acids at N-terminal part of annotated RslR4 (AHL46728.1). Production of RslR4 with 17 extra amino acids resulted in obtaining a soluble protein and this version is referred as RslR4 in the text. This version was used to carry out experimental work.

Generation of *S. bottropensis* Δ*rslO7*.

To inactivate *rslO7*, the suicide plasmid pKGLP2-O7::Am was constructed. For this purpose, *rslO7* gene with approximately 3 kb flanking region was amplified from cos4 using rslO7f (ACCTCGGCGACCTTCAGC) / rslO7r (CTGCCATTGCGGATCCTTTC) primers pair. The PCR product was cloned into pKGLP2 digested with EcoRV, giving pKGLP2-O7. The obtained plasmid was introduced into BW25113 where replacement of the gene coding sequence by an apramycin resistance cassette was accomplished using λ-red mediated recombination technology [2]. Apramycin resistance cassette was amplified with primers pair Am-rslO7-f (CGCGAGGAAAACGTTGAAGGCTCTGGTCACCACCGCCGTGGATATCTCTAGATACCG) and Am-rslO7-r (GGGCTTCGCGGGTACGGCGCACTACGGGCGCCCGGGGTCAAACAAAAGCTGGAGCTC) from pLeere [6]. The obtained plasmid pKGLP2-O7::Am was confirmed by restriction analysis and sequencing, and introduced into wild type *S. bottropensis* by intergeneric conjugation with *E. coli* ET12567 (pUZ8002) harboring the aforementioned construct. The double cross-over mutants were screened for resistance to apramycin and sensitivity to hygromycin, and confirmed by PCR. To generate the marker-free mutant, apramycin resistance cassette was excised by Cre recombinase [5].

Inactivation of *rslO7* resulted in production of new compounds, which were purified and structurally analyzed by NMR and MS. The appearance of alkene derivatives suggests that RslO7 functions as an enoyl reductase in the biosynthesis of the 4-methylpentanoyl-CoA starter unit.

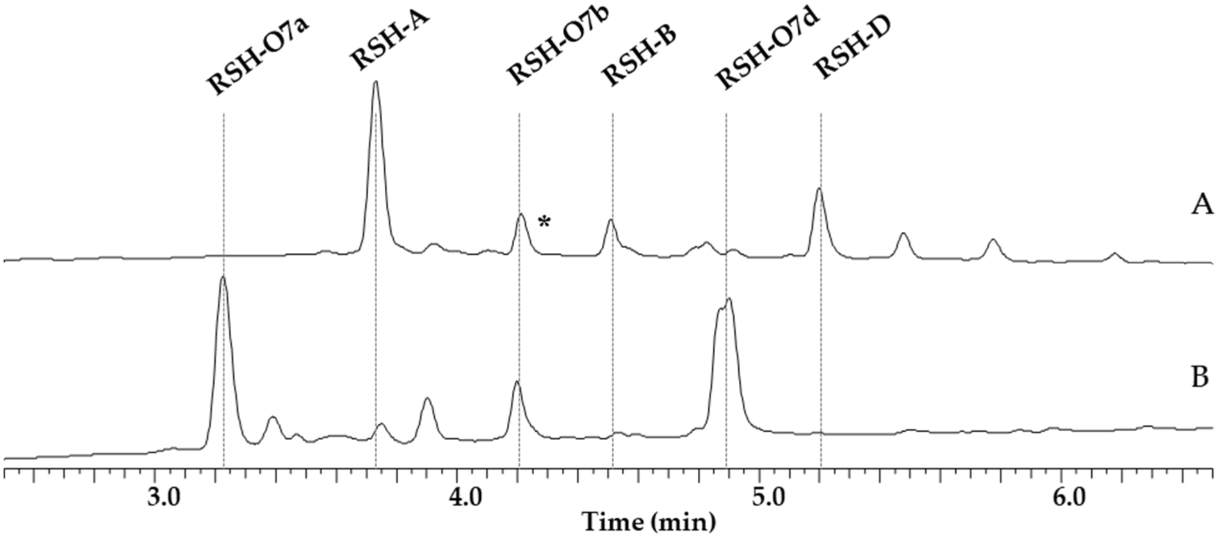

Figure S4. LC-MS analysis of metabolic compounds produced by *S. bottropensis* (A) and *S. bottropensis* Δ*rslO7* (B). * - compound not related to rishirilide biosynthesis.

Isolation and purification of compounds RSHO7a, RSHO7b and RSHO7d from *S. bottropensis* Δ*rslO7*.

A seed culture of *S. bottropensis* ΔrslO7 was grown for 24h in TSB medium and used for inoculation of four liters of YMPGv fermentation culture. After five days of growing, the culture was centrifuged to remove biomass. Then, the supernatant was acidified to pH 4.5 with HCl and exhaustively extracted with ethyl acetate. The collected and evaporated organic phase was dissolved in 40% methanol and fractionated using solid phase extraction column [Oasis HLB 35cc (6g) LP Extraction Cartridge, Waters GmbH, Eschborn, Germany] with subsequent gradient elution with increasing concentrations of methanol. The fraction containing desired compounds (RSHO7a, RSHO7b and RSHO7d) was concentrated *in vacuo* and further purified by semi-preparative HPLC on an Agilent 1100 HPLC system with a DAD detector equipped with a Zorbax SB column (C18, 9.4×150 mm, 5 µm, Agilent Techonologies). The following elution gradient was used: 0 min 40% A, 8 min 0% A, 9 min 0% A, 10 min 40% A, 12 min 40% A (mobile phase A: H_2O with 0.5% CH_3COOH as a solvent modifier and mobile phase B: acetonitrile). The solvents were delivered at 2.5 mL/min.

NMR data for compounds

NMR spectra are recorded using a Varian VNMR-S 600 equipped with 3mm triple resonance inverse and 3mm dual broadband probes. Spectra were measured in CD_3OD at T = 25 °C. Residual solvent signals were used as an internal standard (δ_H = 3.30 ppm, δ_C = 49.0 ppm).

NMR data of compound RSH-O7a (600/150MHz, 25°C, CD_3OD)

Pos.	δ_C [ppm]	δ_H (J Hz) [ppm]	COSY[a]	HMBC[a]
1	199.5			2-H, 9-H, 17-H_3
2	51.5	2.65 q (7.6)	17-H_3	^1J, 17-H_3
3	82.0			2-H, (11-H), 17-H_3
4	81.3			11-H, 12-H
4a	85.0			9-H, 10-H, 11-H
5	157.0			6-H, 7-H, (9-H), 10-H
6	120.7	7.00 dd (8.0, 0.9)	7-H	6-H, 8-H
7	131.2	7.26 dd (8.0, 7.5)	6-H, 8-H	8-H
8	123.9	7.03 dd (7.5, 0.9)	7-H	6-H, 9-H
8a	131.9			7-H, (8-H), 9-H, 10-H
9	139.5	7.62 s		8-H
9a	131.6			9-H, 10-H
10	64.1	5.46 s		(6-H)
10a	122.8			(7-H), 8-H, 9-H, 10-H
11	123.8	5.66 dd (15.7, 1.3)	12-H, (13-H)	13-H, (14-H_3, 15-H_3)
12	144.0	6.14 dd (15.7, 6.9)	11-H, 13-H	11-H, 13-H, 14-H_3, 15-H_3
13	32.6	2.29 ddhept (6.9, 6.8, 1.3)	(11-H), 12-H, 14-H_3, 15-H_3	11-H, 12-H, 14-H_3, 15-H_3
14	22.5	0.98 d (6.8)	13-H	(11-H), 12-H, 13-H, ^1J, 15-H_3
15	22.4	0.96 d(6.8)	13-H	(11-H), 12-H, 13-H, 14-H_3, ^1J
16	177.1			2-H
17	12.3	1.18 d (7.6)	2-H	2-H

[a]weak signals in brackets

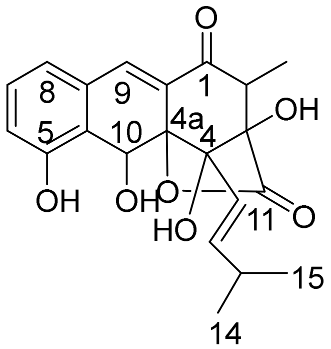

RSH-O7a, (m/z = 385.0, [M-H]$^-$)

NMR data of compound RSH-O7b (600/150MHz, 25°C, CD_3OD)

Pos.	δ_C [ppm]	δ_H (*J* Hz) [ppm]	COSY	HMBC[a]
1	199.3			2-H, 9-H, 10-H, 17-H_3
2	50.5	2.91 q (6.8)	17-H_3	17-H_3
3	85.2			2-H, 17-H_3
4	80.1			2-H, (9-H), 10-H, 11-H, 12-H
4a	139.9			9-H, (10-H), 11-H
5	154.7			7-H, 8-H, (9-H), 10-H
6	111.2	6.91 dd (7.8, 0.9)	7-H	7-H, 8-H
7	128.0	7.31 dd (8.2, 7.8)	6-H, 8-H	(6-H), 8-H
8	121.6	7.47 d (8.2)	7-H, (10-H)	6-H, ^{1}J, 9-H
8a	134.6			7-H, 8-H, 9-H, 10-H
9	127.2	8.38 s	(10-H)	8-H, 10-H
9a	132.0			10-H
10	122.0	8.45 s	(8-H, 9-H)	(9-H)
10a	128.7			6-H, (7-H), 8-H, 9-H
11	132.6	6.19 dd (158, 1.0)	12-H	12-H, 13-H
12	142.5	4.80 dd (15.8, 6.8)	11-H, 13-H	13-H, 14-H_3, 15-H_3
13	32.3	2.22 m	12-H, 14-H_3, 15-H_3	11-H, 12-H, 14-H_3, 15-H_3
14	22.51	0.85 d (6.7)	13-H	(11-H), 12-H, 13-H, ^{1}J, 15-H_3
15	22.49	0.85 d (6.7)	13-H	(11-H), 12-H, 13-H, 14-H_3, ^{1}J
16	175.5			2-H
17	9.6	1.27 d (6.8)	2-H	2-H, ^{1}J

[a]weak signals in brackets

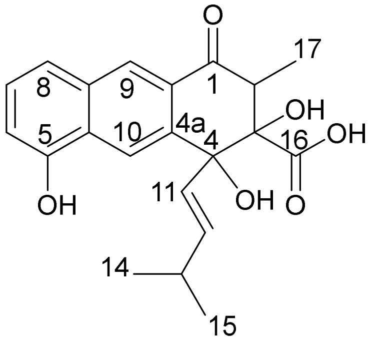

RSH-O7b, (m/z = 369.1, $[M-H]^-$)

NMR data of compound RSH-O7d (600/150MHz, 25°C, CD_3OD)

Pos.	δ_C [ppm]	δ_H (*J* Hz) [ppm]	COSY	HMBC[a]
1	197.6			2-H, 9-H, 17-H_3
2	52.9	2.97 q (6.5)	17-H_3	17-H_3
3	79.9			2-H, 11-H, 17-H_3
4	135.3			2-H, (9-H), 11-H, 12-H
4a	115.6			9-H, 11-H, 12-H
5	155.6			6-H, (7-H), 8-H, 9-H
6	113.3	6.85 dd (7.6, 0.9)	7-H, 8-H	7-H, 8-H
7	129.7	7.34 dd (8.2, 7.6)	6-H, 8-H	^1J
8	122.3	7.39 dd (8.2, 0.9)	6-H, 7-H	6-H, 9-H, ^1J
8a	136.7			7-H, 8-H, 9-H
9	121.0	7.98 s		8-H, ^1J
9a	128.9			9-H
10	150.8			8-H, 9-H, 11-H, 12-H
10a	117.4			6-H, 7-H, 8-H, 9-H
11	120.1	6.19 d (2.5)	12-H	12-H, 13-H
12	84.0	5.09 dd (4.8, 2.5)	11-H, 13-H	11-H, 13-H, 14-H_3, 15-H_3
13	34.0	2.24 m	12-H, 14-H_3, 15-H_3	11-H, 12-H, 14-H_3, 15-H_3
14	17.6	1.13 d (6.8)	13-H	12-H, 13-H, 15-H_3
15	18.7	1.17 d (6.8)	13-H	12-H, 13-H, 14-H_3
16	174.3			2-H
17	8.9	1.30 d (6.8)	2-H	2-H, ^1J

[a]weak signals in brackets

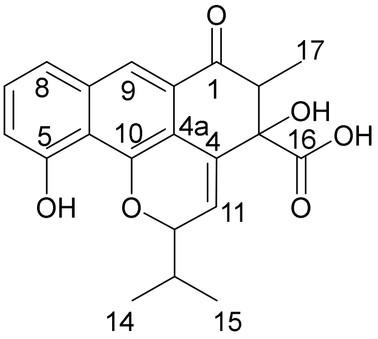

RSH-O7d, (m/z = 367.1, $[M-H]^-$)

^{1}H NMR of RSH-O7a (600MHz, CD_3OD, 25 °C)

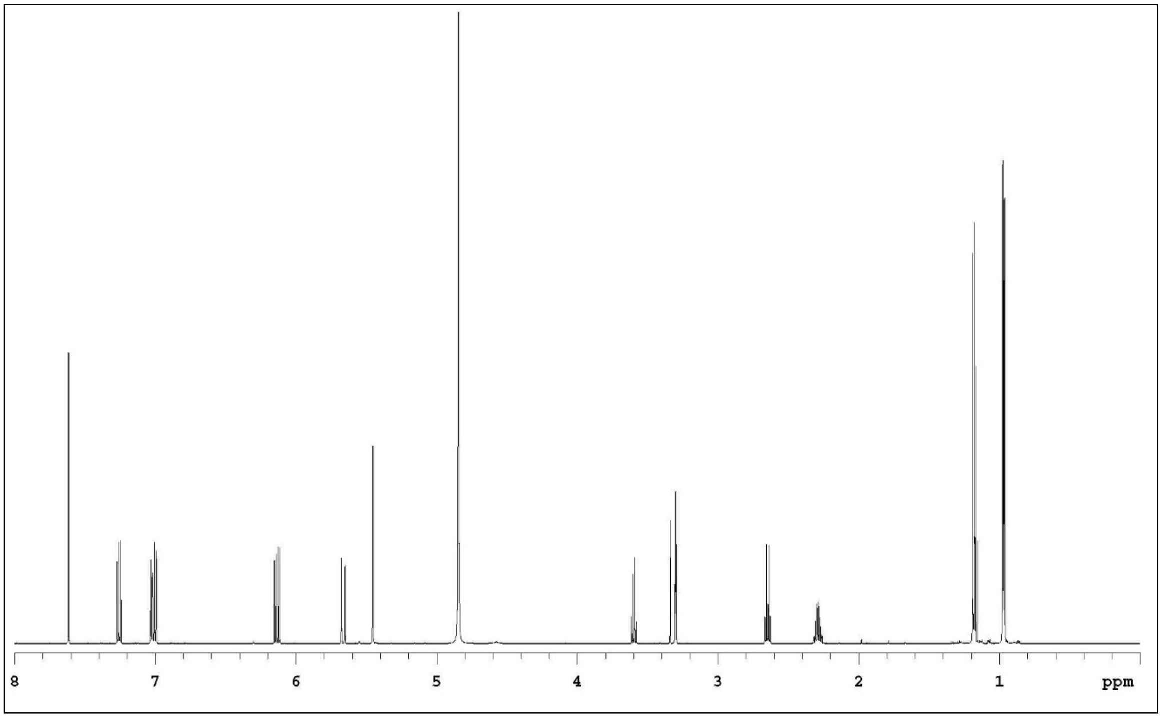

^{13}C NMR of RSH-O7a (150MHz, CD_3OD, 25 °C)

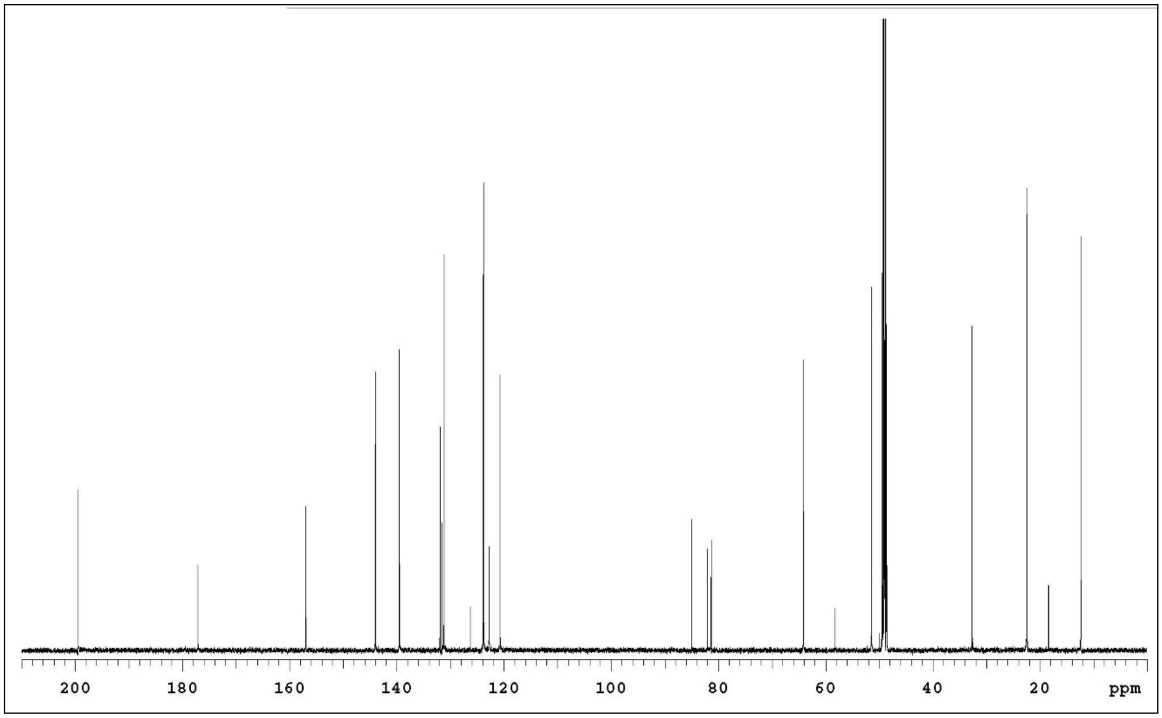

COSY of RSH-O7a (600MHz, CD_3OD, 25 °C)

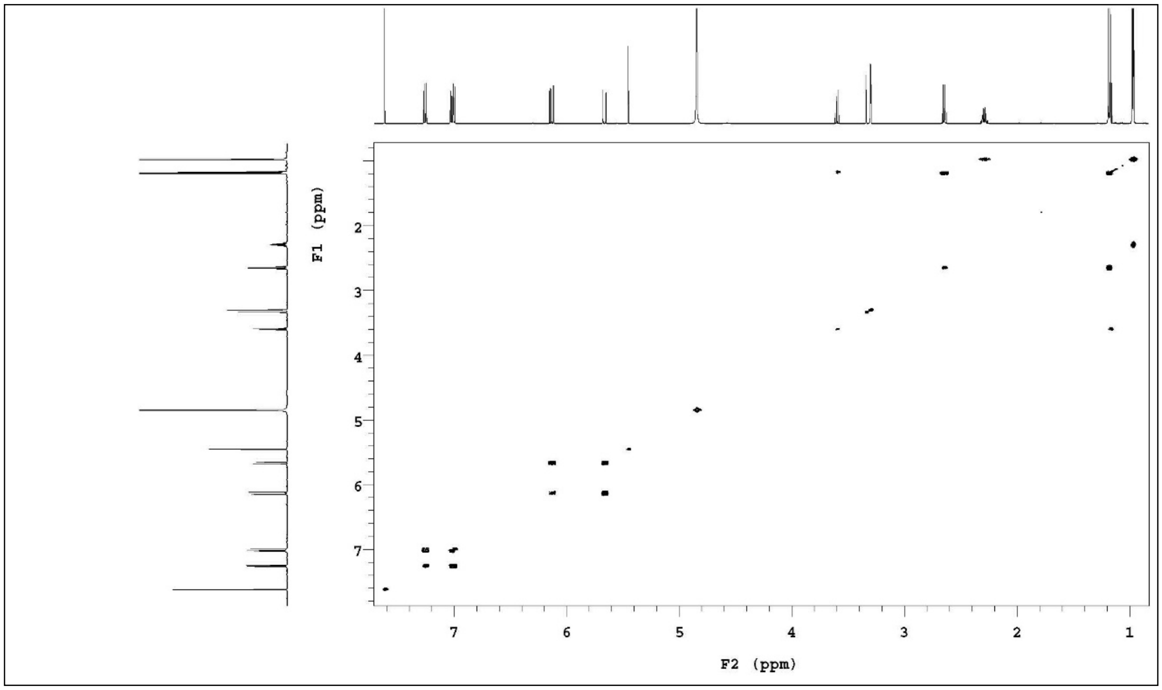

HSQC of RSH-O7a (600MHz, CD_3OD, 25 °C)

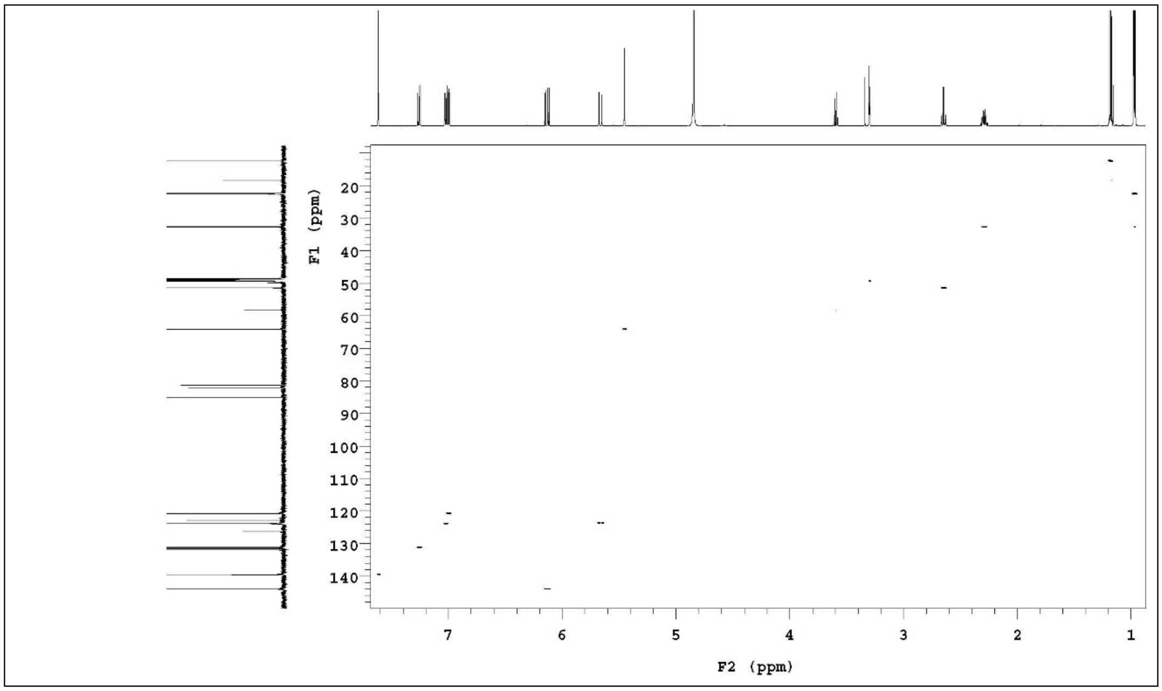

HMBC of RSH-O7a (600MHz, CD_3OD, 25 °C)

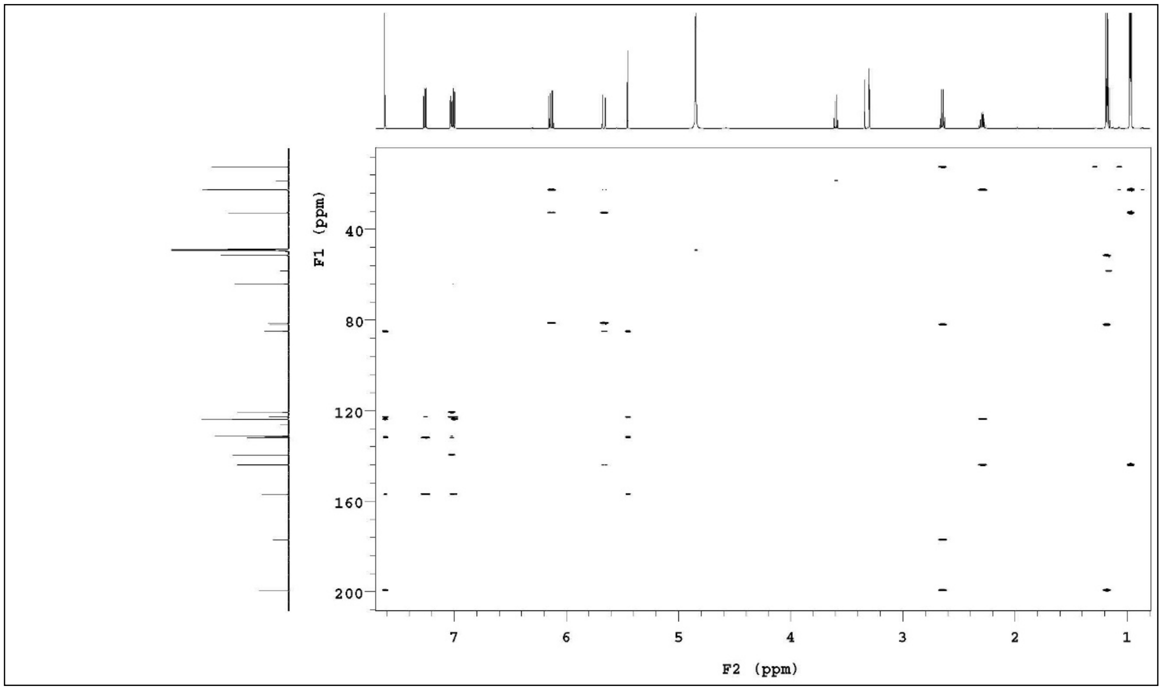

^{1}H NMR of compound RSH-O7b (600MHz, 25°C, CD_3OD)

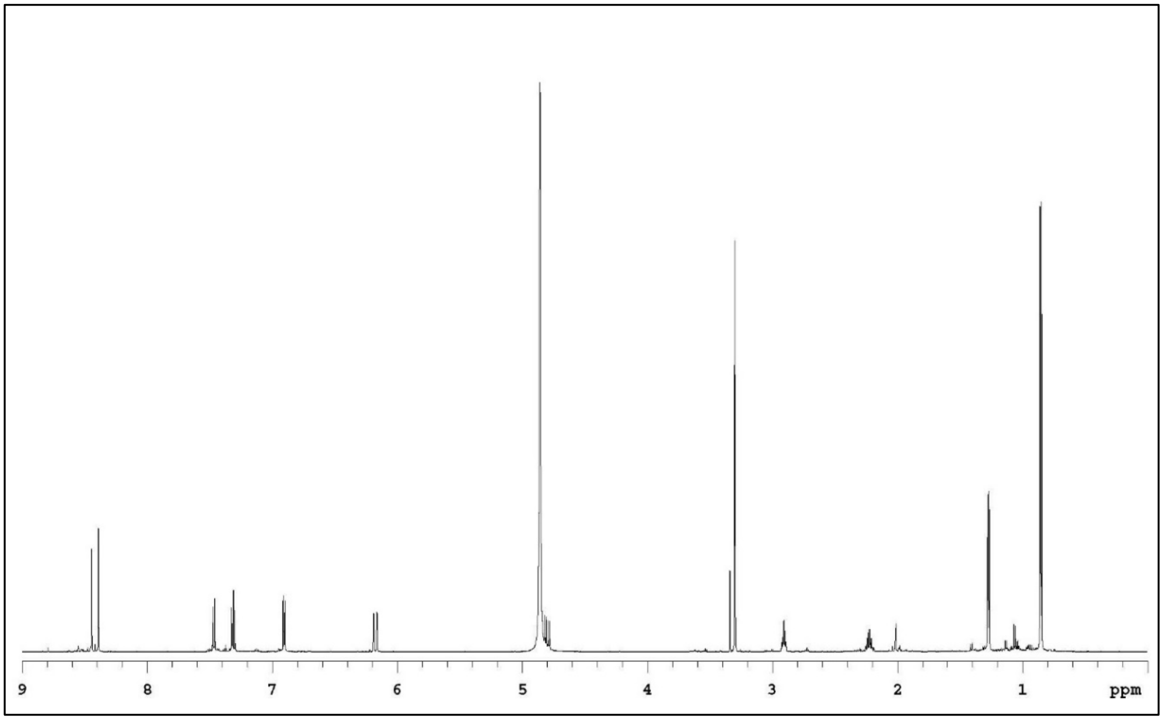

^{13}C NMR of compound RSH-O7b (150MHz, 25°C, CD_3OD)

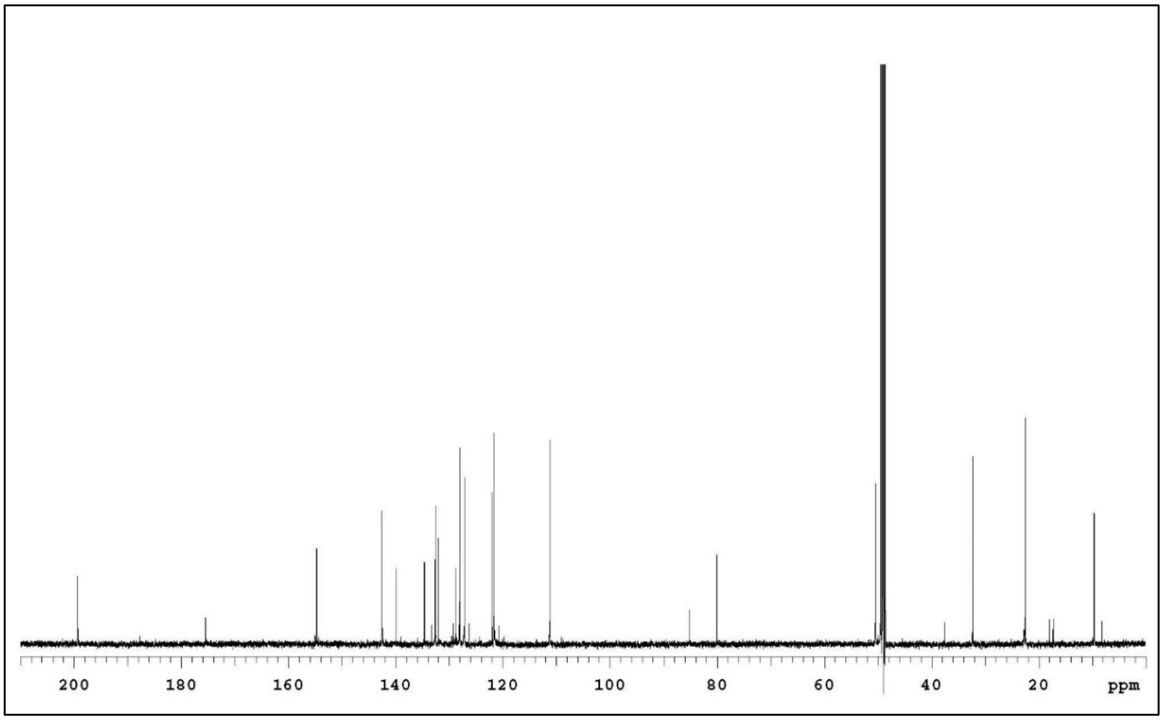

COSY NMR of compound RSH-O7b (600MHz, 25°C, CD_3OD)

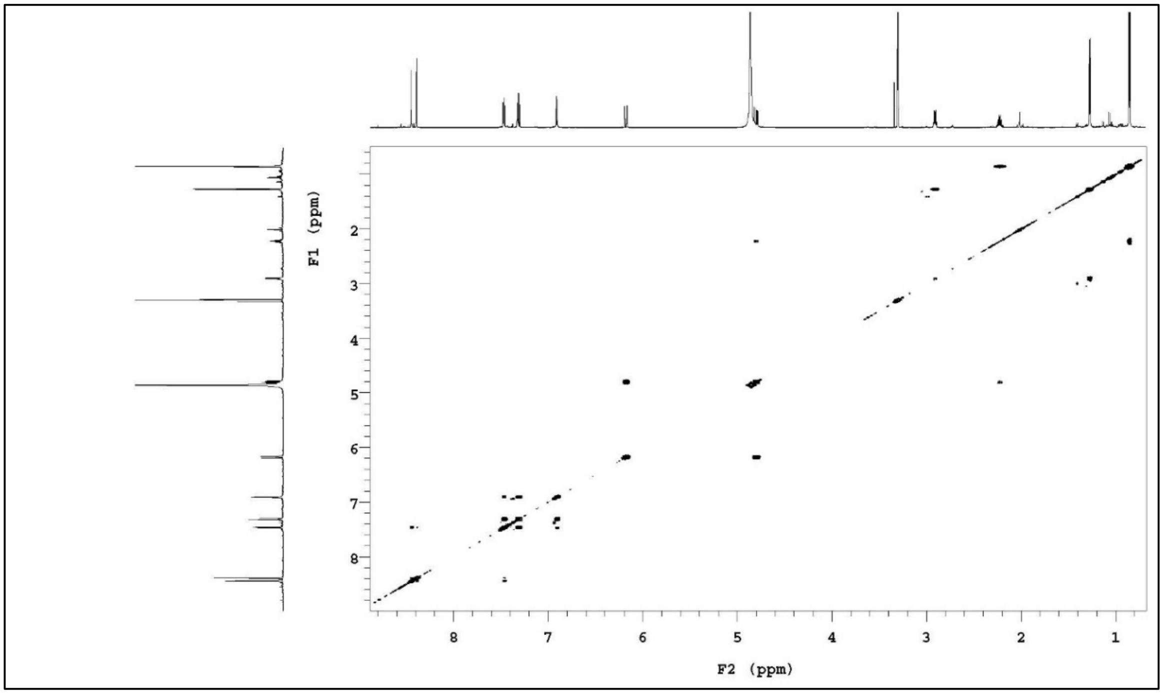

HSQC NMR of compound RSH-O7b (600MHz, 25°C, CD_3OD)

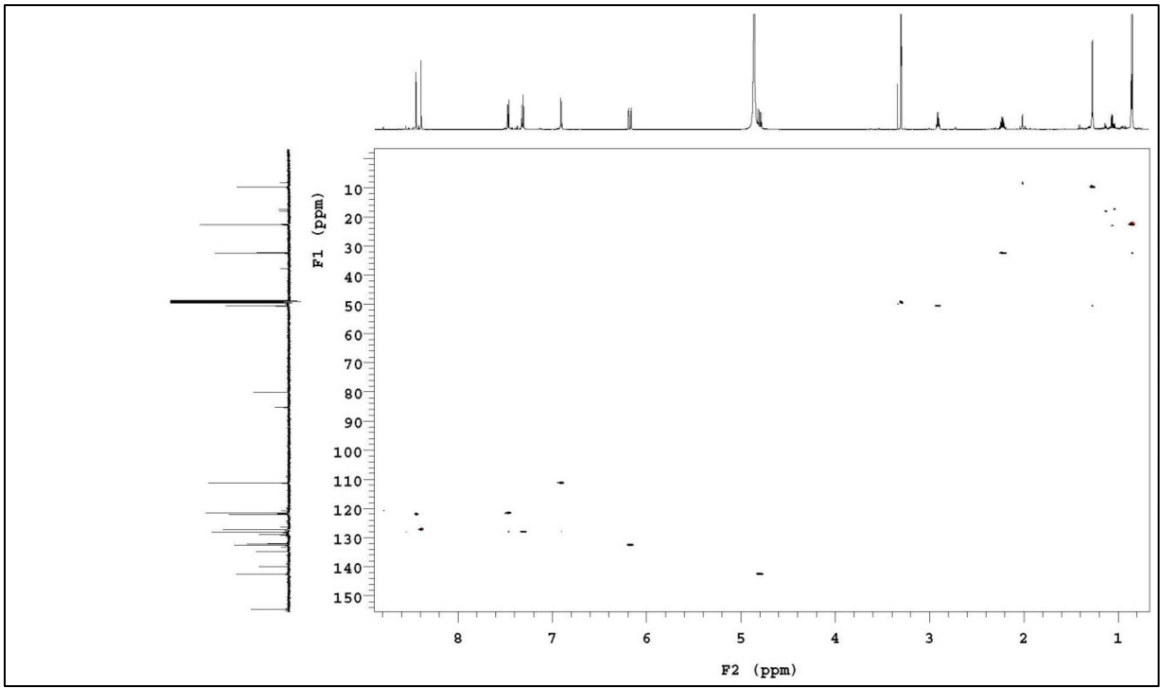

HMBC NMR of compound RSH-O7b (600MHz, 25°C, CD_3OD)

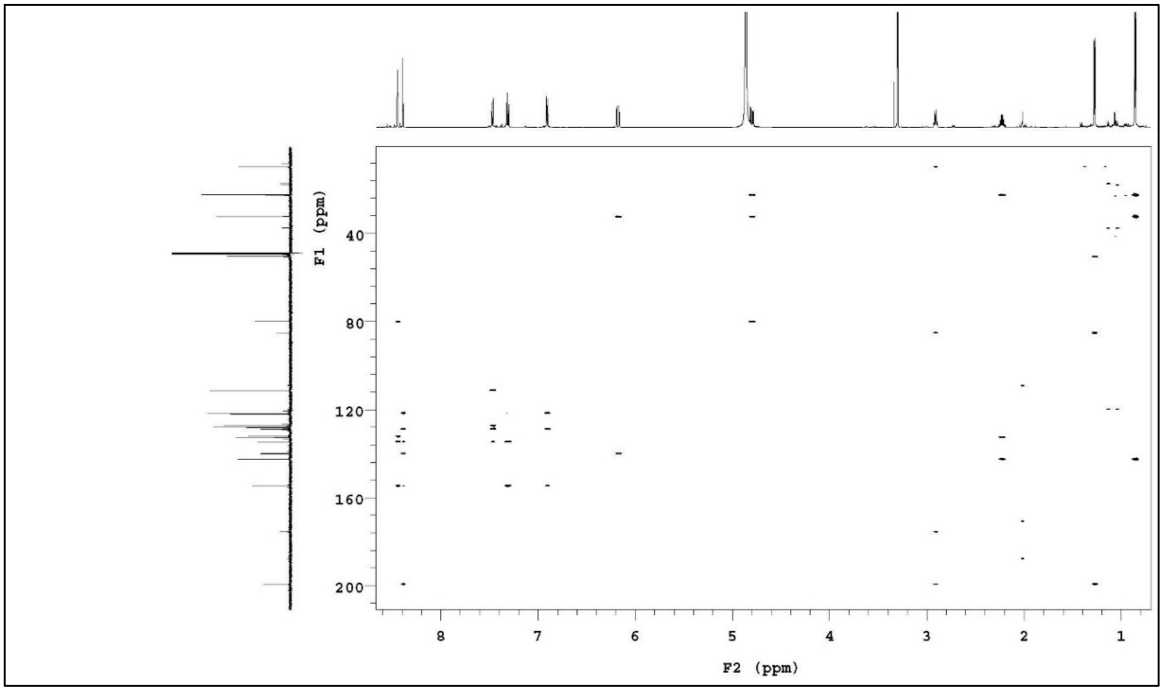

^{1}H NMR of compound RSH-O7d (600MHz, 25°C, CD_3OD)

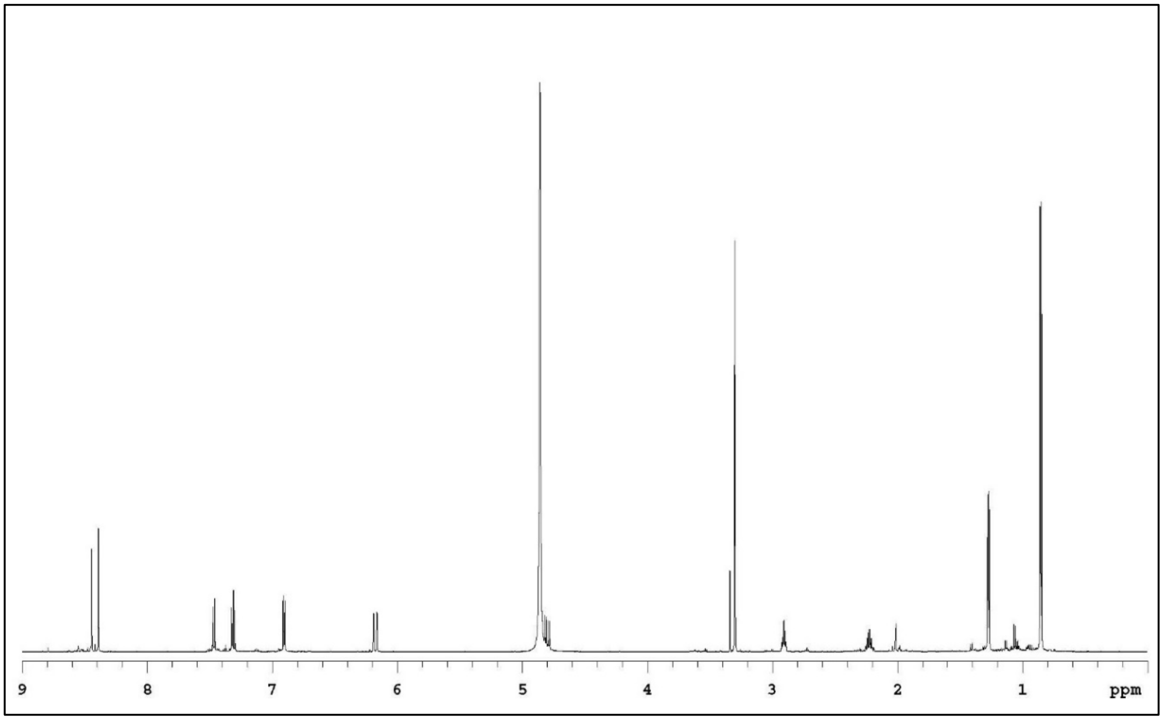

^{13}C NMR of compound RSH-O7d (150MHz, 25°C, CD_3OD)

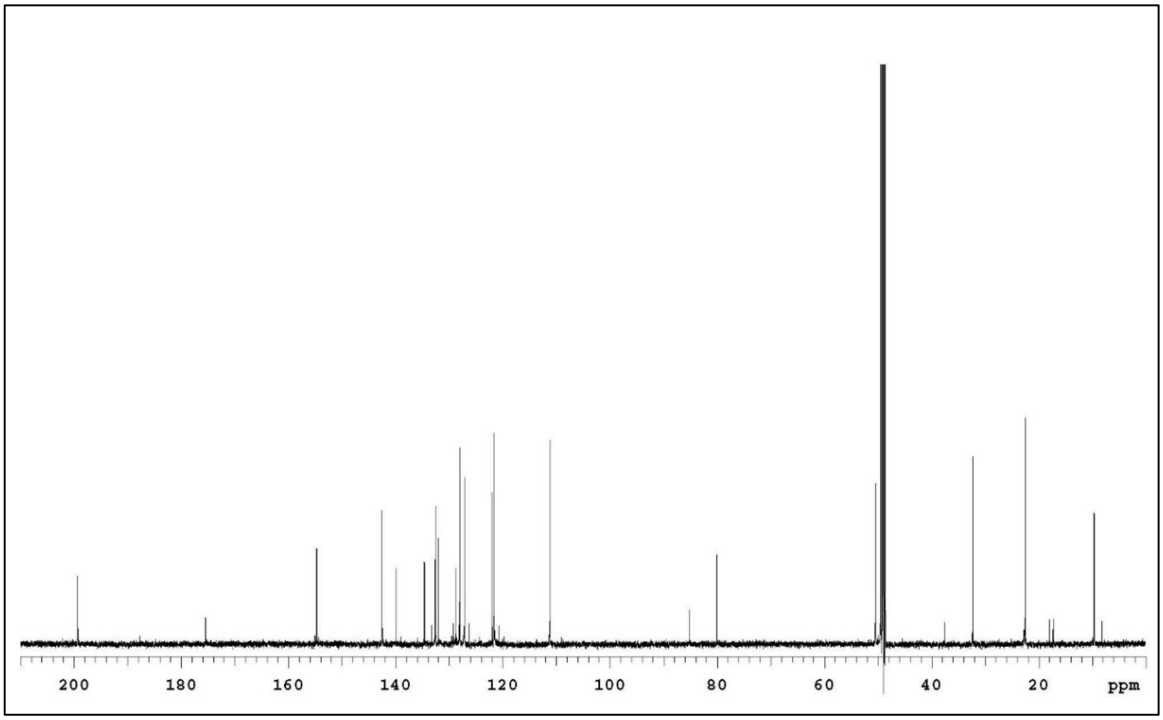

COSY NMR of compound RSH-O7d (600MHz, 25°C, CD_3OD)

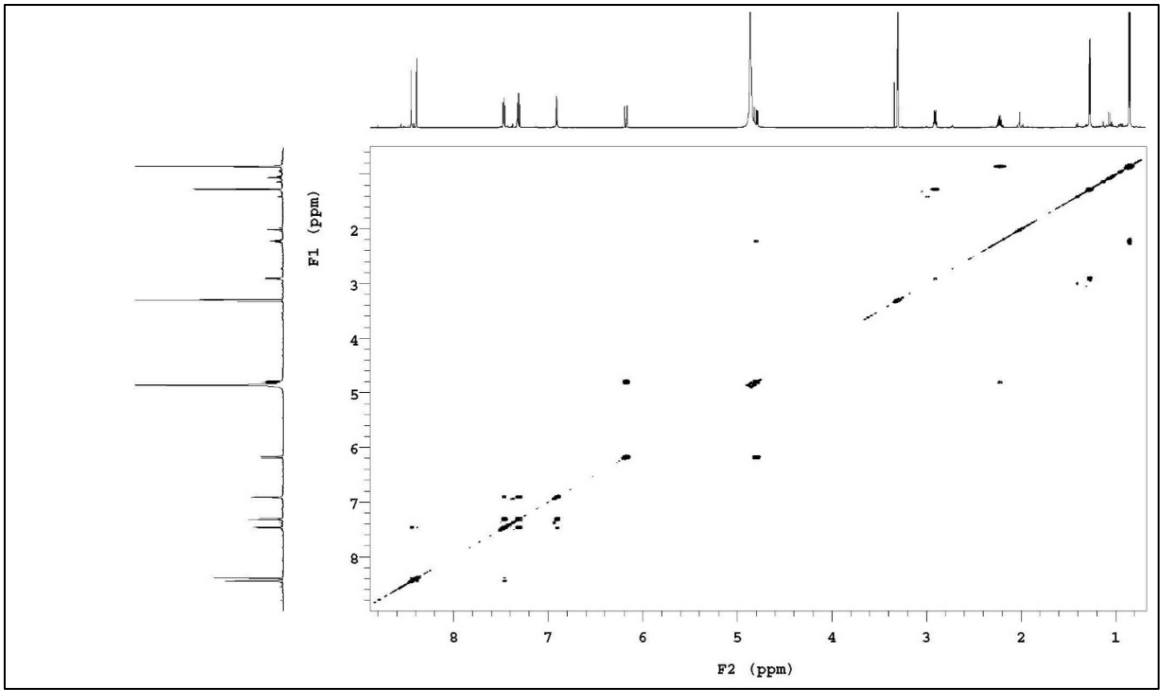

HSQC NMR of compound RSH-O7d (600MHz, 25°C, CD_3OD)

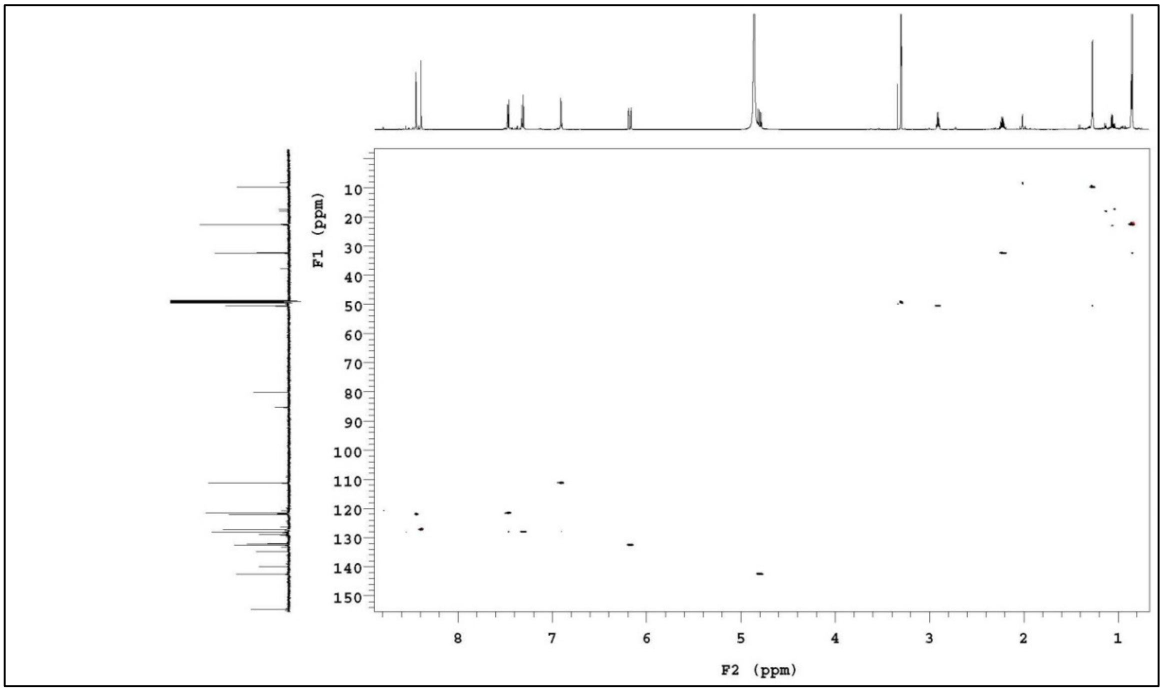

HMBC NMR of compound RSH-O7d (600MHz, 25°C, CD_3OD)

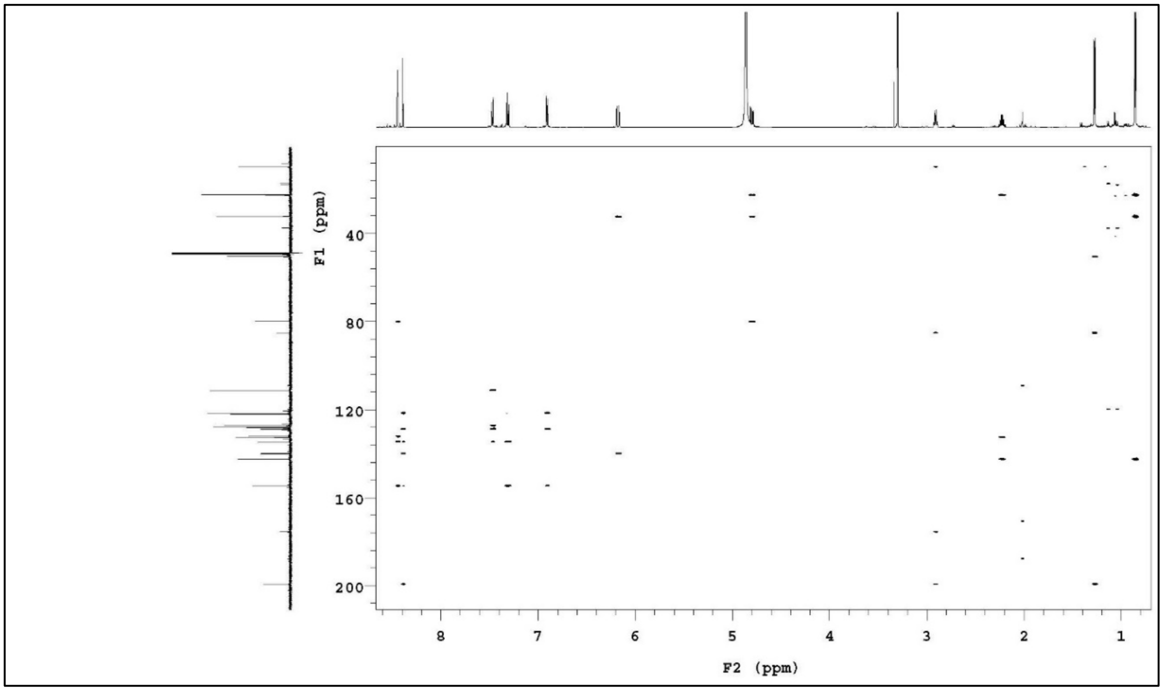

References

1. Yan, X.; Probst, K.; Linnenbrink, A.; Arnold, M.; Paululat, T.; Zeeck, A.; Bechthold, A. Cloning and heterologous expression of three type II PKS gene clusters from *Streptomyces bottropensis*. *ChemBioChem* **2012**, *13*, 224–230.
2. Gust, B.; Chandra, G.; Jakimowicz, D.; Yuqing, T.; Bruton, C. J.; Chater, K. F. Lambda red-mediated genetic manipulation of antibiotic-producing Streptomyces. *Adv. Appl. Microbiol.* **2004**, *54*, 107–128.
3. Bierman, M.; Logan, R.; O'Brien, K.; Seno, E.T.; Rao, R.N.; Schoner B.E. Plasmid cloning vectors for the conjugal transfer of DNA from *Escherichia coli* to *Streptomyces spp*. *Gene* **1992**, *116*, 43–49.
4. Herrmann, S.; Siegl, T.; Luzhetska, M.; Petzke, L.; Jilg, C.; Welle, E.; Erb, A.; Leadlay, P.F.; Bechthold, A.; Luzhetskyy, A. Site-specific recombination strategies for engineering actinomycete genomes. *Appl. Environ. Microbiol.* **2012**, *78 (6)*, 1804–1812.
5. Fedoryshyn, M.; Welle, E.; Bechthold, A.; Luzhetskyy, A. Functional expression of the Cre recombinase in actinomycetes**.** *Appl. Microbiol. Biotechnol.* **2008**, *78 (6)*, 1065–1070.
6. Tsypik, O.; Makitrynskyy, R.; Bera, A.; Song, L.; Wohlleben, W.; Fedorenko, V.; Ostash, B. Role of GntR family regulatory gene *SCO1678* in gluconate metabolism in *Streptomyces coelicolor* M145. *BioMed Res. Internat.* **2017**, 9529501, p.9.
